# Management of Diabetes in Pregnancy: A Review of Clinical Guidelines and Practices

**DOI:** 10.7759/cureus.79334

**Published:** 2025-02-19

**Authors:** Raniah A Albairmani, Basheer M Basheer, May M Macky, Tala Al Syouti, Haya AlZubaidy, Eyman Elfaki, Alweena Kidwai, Yousif M Basheer, Fatma Ahmed, Mona Salaheldin

**Affiliations:** 1 Medicine, HMS (Health and Medical Services) Al Garhoud Hospital, Dubai, ARE; 2 Medicine, HMS (Health and Medical Services) Mirdif Hospital, Dubai, ARE; 3 Medicine, Dr. Sulaiman Al Habib Hospital, Dubai, ARE; 4 Internal Medicine, Ajman University, Ajman, ARE; 5 Endocrinology, Diabetes and Metabolism, HMS (Health and Medical Services) Al Garhoud Hospital, Dubai, ARE

**Keywords:** continuous glucose monitoring, diabetes, fetal outcomes, gestational diabetes mellitus, pregnancy, tight glycemic control

## Abstract

This literature review assesses clinical guidelines for pre-existing diabetes and gestational diabetes mellitus (GDM) in the areas of diagnosis, management, and maternal-fetal outcomes. A structured search was conducted across PubMed and Google Scholar, supplemented by targeted screening of guideline repositories from the American Diabetes Association (ADA), National Institute for Health and Care Excellence (NICE), and World Health Organization (WHO). Included studies and guidelines were selected based on relevance to diagnosis, therapeutic strategies, or maternal-neonatal outcomes, with exclusion criteria applied to non-English publications and non-clinical recommendations. A comparative analysis of guidelines from the ADA, NICE, and WHO was performed to evaluate prevalence, therapeutic approaches, and postpartum management. Early diagnosis, stringent blood glucose control, and multidisciplinary care with the aim to avoid macrosomia, congenital abnormalities, and neonatal hypoglycemia guide the management guidelines. Glycated hemoglobin (HbA1c) (<6.5%) optimization and supplementation with folic acid are critically required prior to conception in all women with previously diagnosed diabetes. Continuous glucose monitoring (CGM) and insulin pump therapy are valued but burdened by availability and access constraints. A postpartum visit with 75 g oral glucose tolerance test (OGTT) at 4-12 weeks is essential for the detection of persistent diabetes. Variation of diagnostic criteria among guidelines reflects the requirement for standardization. Expansion of coverage by insurance for CGM and preconception care is important for providing equal access. The cost-effectiveness of new technologies and health disparities in low-resource settings must be addressed in future research.

## Introduction and background

Diabetes in pregnancy is a significant public health issue with dire implications for maternal and fetal health and considerable economic expenditure in terms of complications like preterm labor and neonatal intensive care. Its control requires stringent glycemic regulation to limit adverse effects. Diabetes can be classified broadly into two categories: pre-gestational diabetes, which involves type 1 and type 2 diabetes diagnosed before pregnancy, and gestational diabetes mellitus (GDM), which emerges during pregnancy and corrects after delivery. Global GDM prevalence is 2-10%, with low- and middle-income countries having a higher prevalence due to inadequate access to health care. GDM is present in 15-25% of pregnancies in the UAE, where it is driven by obesity and genetic susceptibility, while pre-existing diabetes is seen in 1-3% of pregnancies [1].

The prevalence of GDM significantly varies depending on population and diagnostic criteria and has been estimated to affect between 2% and 10% of pregnancies worldwide [2]. The risk for both maternal and fetal complications is increased with both pre-existing and gestational diabetes. Maternal risks include preeclampsia, cesarean delivery, and worsening of pre-existing diabetes. In contrast, fetal risks include macrosomia, birth defects, preterm birth, neonatal hypoglycemia, and long-term risks such as obesity and type 2 diabetes mellitus (T2DM) later in life [3]. 

This review compares the guidelines on diabetes management in pregnancy such as preconception care, screening, and therapeutic interventions, emphasizing the need for early intervention and health equity.

## Review

Preconception period

Preconception care is essential for reducing pregnancy complications in women with diabetes. For those with pre-existing diabetes, maintaining glycated hemoglobin (HbA1c) <6.5% before conception can lower the risk of congenital anomalies by up to 70% [1]. Also, the goal is to convert from teratogenic medication, such as angiotensin-converting enzyme (ACE) inhibitors, to insulin safely, attain the best possible control, and minimize risk while changing medication [3]. It has been seen that women with a family history of diabetes, a history of GDM, or a history of having given birth to a macrosomic baby are at higher risk of developing GDM, and thus early intervention is critical [4]. In addition, pre-existing diabetes in women is complicated by aggravated retinopathy, nephropathy, hypertension, and preeclampsia. Screening for complications and cardiovascular status is, thus, most critical [5]. Pre-existing diabetes in women carries a high risk of neural tube defects (NTDs) compared to women without diabetes. High evidence is available for low-dose folate use to prevent NTDs and perhaps other congenital defects. Women with diabetes are recommended an increased dose of folate, with a total daily intake of up to 5 mg from three months prior to conception. This must be incorporated into an individualized preconception care plan, as required for the patient, to optimize maternal and fetal well-being and reduce risks of pregnancy complications [6]. Despite the evidence, preconception counseling is only given to 30-50%, highlighting care shortcomings [7].

Screening for diabetes in early pregnancy

The incidence of T2DM among women of childbearing age is rising. Since T2DM often presents without symptoms in its initial stages and women with significant hyperglycemia early in pregnancy face a higher risk of adverse outcomes, early detection and treatment of diabetes are paramount. As a result, most international associations such as the International Association of Diabetes and Pregnancy Study Groups (IADPSG), the American Diabetes Association (ADA), and the World Health Organization (WHO) recommend therefore to screen for overt diabetes at the first antenatal visit using an FPG, HbA1c, or 75 g oral glucose tolerance test (OGTT) with the same cut-offs as for non-pregnant populations. Anyone above the cut-off is considered high risk by interpretation. To confirm a diagnosis of overt diabetes, FPG and HbA1c measurements should ideally be repeated twice. While HbA1c is useful for screening diabetes, its lower sensitivity in pregnancy, due to erythrocyte turnover, limits its utility for GDM [8].

Clinical guidelines of GDM: overview

Clinical guidelines on the management of diabetes in pregnancy have been developed by several prestigious professional associations like ADA, the International Diabetes Federation (IDF), the National Institute for Health and Care Excellence (NICE), and WHO. These guidelines are based on evidence from a variety of sources, including randomized controlled trials, observational studies, and expert consensus. These are regularly updated to include new scientific evidence and advances in clinical practice. Specifically, their guidelines are developed to enhance maternal and fetal outcomes and ensure optimal management of diabetes throughout pregnancy. Their guidelines also stress the need for a multidisciplinary team to manage diabetes in pregnant women, which may include obstetricians, endocrinologists, dietitians, and diabetes educators [9].

GDM is a common complication of pregnancy, and its usual method of diagnosis is screening for glucose intolerance. The most widely used method is the OGTT. Both ADA 2023 and NICE 2022 recommend universal screening for GDM between 24-28 weeks of gestation with a 75 g OGTT [10]. However, high-risk populations such as those with a BMI of 30 or more, a family history of diabetes, and a prior history of GDM or macrosomia should undergo screening earlier than 15 weeks of gestation to detect potential glucose intolerance before it worsens [11]. The guidelines of the ADA (2023) recommend the 75 g OGTT with the following cut-off points for diagnosis: fasting glucose ≥92 mg/dL (5.1 mmol/L), a one-hour level of ≥180 mg/dL (10 mmol/L), and a two-hour level of ≥153 mg/dL (8.5 mmol/L). Moreover, WHO’s diagnostic thresholds include fasting glucose ≥92 mg/dL and a two-hour ≥153 mg/dL [11].

Rising GDM incidence correlates with increasing maternal age and obesity. Discrepancies in criteria (e.g., IADPSG vs. NICE) contribute to prevalence variability. Despite these minor variations in the diagnostic criteria, early detection and intervention have been agreed upon by both societies as the most crucial factor in the management of GDM [12]. Early detection of the condition allows timely treatment to be instituted that can diminish the risks of maternal complications such as preeclampsia, cesarean delivery, and worsening of pre-existing diabetes, and fetal complications such as macrosomia, birth defects, and neonatal hypoglycemia [10]. Further, it is also emphasized in some recommendations that children born to women with GDM have long-term risks of obesity and later development of T2DM, thus requiring follow-up care for many years after delivery [13]. Moreover, children born to women with GDM can inherit type 1 diabetes despite glycemic control [14].

Management of pre-existing diabetes

Pre-existing diabetes control consists of optimizing HbA1c before conception, throughout pregnancy, and after delivery in order to prevent congenital malformations and stillbirth as well as for screening complications and education on hypoglycemia. Strict glycemic control is especially important to improve pregnancy outcomes and decrease maternal and fetal complications since uncontrolled blood glucose is dangerous in terms of causing maternal and fetal adverse outcomes such as preeclampsia, macrosomia, and birth defects [15]. The ADA (2023) also recommended folic acid supplementation to reduce the risk of neural tube defects, and tests for cardiovascular health, renal function, and diabetic complication screening like retinopathy, nephropathy, and neuropathy [16]. The ADA and NICE-recommended targets are the preprandial blood glucose levels between 70-95 mg/dL (3.9-5.3 mmol/L) and less than 140 mg/dL (7.8 mmol/L) one hour after meals [17].

It must be noted that early pregnancy hyperglycemia is associated with an increased risk of congenital malformations, while hyperglycemia later in pregnancy results in fetal overgrowth (macrosomia) and other complications such as shoulder dystocia during delivery [18]. Insulin treatment remains the mainstay of treatment in most women with established diabetes. Whereas oral medications like metformin and glyburide are potentially available to use, the drugs are infrequently utilized in pregnancy, specifically pre-gestational status, due to potential issues of safety and fetal welfare, especially in the first trimester. Basal-bolus insulin therapy can be included and adjusted to fit the patient, whose needs might vary during the pregnancy as with increased insulin resistance. The management plan must be comprehensive, for example, regular blood glucose monitoring (self-monitoring of blood glucose (SMBG)) to guide insulin adjustments to maintain the target range of glucose [9]. 

Continuous Glucose Monitoring (CGM) 

CGM is becoming increasingly popular in diabetes management in pregnancy because it can potentially provide real-time data and prevent hyperglycemia and hypoglycemia. CGM decreases hypoglycemia and HbA1c by 0.5% compared to SMBG [19]. CGM is not without issues, including barriers to use, with about 40% of the patients being non-adherent, concerns about fear of hypoglycemia, and expense, with the cost being about $300 per month for the uninsured. Such patients require frequent antenatal follow-ups for medication dose adjustment, checking of maternal health, and evaluation of fetal growth, preferably with ultrasound for checking for signs of macrosomia or other pathology. Regular screening should also be performed for complications like preeclampsia, diabetic ketoacidosis, or urinary tract infection so that early intervention may be done. Multidisciplinary teamwork needs to be given due attention by obstetricians, endocrinologists, dietitians, and other healthcare providers in order to take care of and deal with the complex needs of pregnant women with pre-existing diabetes [9].

Insulin Pump Therapy

An RCT in 2017 established that insulin pumps improve glycemic control in pregnant women with type 1 diabetes. It showed that women receiving insulin pumps had much better HbA1c levels, fewer severe hypoglycemic events by 35%, and fewer rates of neonatal hypoglycemia compared to women receiving insulin injections [20]. Moreover, fewer neonatal intensive care unit (NICU) admissions were experienced. These studies affirm the benefits of insulin pumps in achieving optimal glucose control and reducing undesirable outcomes. Insulin pumps, although beneficial, require careful monitoring and pose some drawbacks, including a risk of infection due to inadequate site rotation and limited access or coverage, particularly in low-resource settings where cost is likely to be a significant barrier [21].

Pregnant women on insulin pumps should have good hygiene practices, such as frequent site rotation of the infusion, to minimize such risks. Additionally, technical problems in insulin pumps, such as malfunction or failure of the pump, can cause interruptions in insulin infusion. If the pump fails, the patient will be required to have a backup, normally reverting to a subcutaneous infusion of insulin. It is here that patient education on how to navigate such a situation becomes useful. The cost of insulin pumps may also be a major constraint in areas where insurance does not cover the pump or the supplies for it. Accessibility of insulin pumps is also restricted in some countries and healthcare systems; thus, not all pregnant women with diabetes will be able to use them. 

Monitoring of Complications

Besides optimization of insulin delivery, pregnant women with existing diabetes need close monitoring of complications such as hypertensive disorders, diabetic ketoacidosis (DKA), and fetal growth anomalies that can threaten the life of both mother and baby [22]. It has been established that regular ultrasounds need to be done in the second and third trimesters to monitor fetal development and overall status. The mother's hyperglycemic state causes fetal abnormalities of pregnancy; glucose is transferred from the mother to the fetus through the placenta, resulting in macrosomia, intrauterine growth restriction, congenital anomalies, preterm birth, and neonatal hypoglycemia in the newly born infants [23]. The severity of such complications has a close relation to the control of maternal glycemia, with the highest risk to both mother and fetus if diabetes is inadequately controlled. Furthermore, maternal complications of preeclampsia, cesarean section, and worsening of underlying diabetes can complicate the pregnancy [24]. Proper glucose control throughout pregnancy is greatly important and helps promote maternal and fetal outcomes by minimizing the risks mentioned above.

Fetal complications

Diabetic gestation sequelae in the fetus are many and may have severe effects on the baby's short- and long-term health. One of the most common and severe short-term sequelae is macrosomia, birth weight greater than 4000-4500 grams, with a 26% incidence rate [25]. It occurs as a result of hyperglycemia in the maternal circulation, which crosses the placenta and leads to fetal hyperinsulinemia. Because of this, the fetus is overgrown and may suffer several birth injuries, such as shoulder dystocia (3-12% rate of incidence), brachial plexus injury, and fractures of the clavicle [26]. Furthermore, macrosomic neonates are prone to neonatal hypoglycemia (25% incidence rate) since the intrauterine hyperglycemic environment leads their pancreas to continue releasing excess insulin, and their blood glucose can fall sharply once glucose transfer from the placenta is stopped after birth. In the long term, in-utero exposure to high blood glucose levels is linked to a 1.5-fold higher risk of obesity in children and a 7.4-fold higher risk of T2DM [27,28].

Respiratory distress syndrome also happens more often in infants of mothers with diabetes, especially when maternal blood glucose control is inadequate. The reason for this is impaired surfactant production within the lungs which causes the newborn to breathe poorly [29]. Congenital malformations are another significant risk in newborns of women with poorly controlled pre-existing diabetes, particularly if blood glucose is elevated in the first trimester when organogenesis occurs. Uncontrolled maternal blood glucose levels interfere with normal fetal growth, creating structural defects. A few congenital anomalies seen in pregnancies complicated by maternal hyperglycemia are cardiac malformations like ventricular septal defects, neural tube defects like spina bifida, renal malformations like renal agenesis, and osseous dysplasias like caudal regression syndrome. Optimal glucose control during the pre-conceptual period and in the first trimester of pregnancy has been shown to significantly reduce the risk of such anomalies [30]. In addition, both pre-existing diabetes and GDM have been associated with an increased risk of stillbirth, particularly with poor control of maternal blood glucose. Hyperglycemia also causes placental insufficiency, which damages the blood and oxygen supply to the fetus and generates increased risks of fetal mortality. Studies show that tight regulation of blood glucose in pregnancy can reduce the risk of stillbirth [31].

This risk does not diminish once gross birth defects and complications in children of diabetic women, poorly controlled in this case, have been avoided; children are at lifetime risk for disease, including obesity as a child and T2DM. It has been suggested that the following heightened risk is due to the intrauterine environment, with fetal overinsulin production and abnormal growth patterns setting the stage for later metabolic malfunction [32]. Thus, good diabetic control during pregnancy is not only required to reduce the short-term risks of delivery but also to reduce the subsequent chronic disease burden in the infant. In conclusion, prevention of these serious fetal complications of diabetes relies on excellent maternal blood glucose control along with full prenatal care.

Management of GDM

GDM normally reverses after the gestational period. However, the disease dramatically increases the chance of T2DM in the later years. Main management priorities in GDM involve attempts toward achieving normoglycemia to prevent complications for the mother and the fetus. The first line of managing GDM involves changes in lifestyle such as nutrition, in addition to physical activities if allowed by the gynecologist. These can include a balanced diet with low-glycemic index meals consisting of 40% carbohydrates, 30% protein, and 30% fats, with carbohydrate counting to help prevent hyperglycemia, as stated by ADA (2023), and NICE (2022), and regular physical activities such as brisk walking for 150 minutes per week if recommended by the gynecologist to improve insulin sensitivity [33]. 

However, in some women, lifestyle interventions by themselves often are inadequate for optimum glycemic control. In cases when glycemic targets cannot be achieved through diet and exercise, pharmacologic therapy is instituted. Insulin, whether neutral protamine hagedorn (NPH) or analogs, remains the first-line treatment, as it does not cross the placenta and has a long-established safety record [9]. Oral medications, such as metformin and glyburide, may be considered if insulin is refused. However, it has been associated with preterm birth [34]. The ADA (2023) recommends starting insulin therapy when fasting blood glucose is above 95 mg/dL (5.3 mmol/L) or when postprandial glucose levels are above 140 mg/dL (7.8 mmol/L) [8]. This requires close monitoring of blood glucose to adjust insulin dosing and avoid hypoglycemia and hyperglycemia. 

Besides careful maternal blood glucose management, the fetus must also be followed closely. Regular SMBG is recommended to ensure that maternal glucose levels stay within target ranges. Fetal surveillance, including routine ultrasounds every four weeks and a fetal echocardiogram if first-trimester hyperglycemia was present, is particularly important in cases of poor glycemic control or suspected macrosomia, as these conditions increase the risk of complications [35]. Given that macrosomic newborns are more likely to suffer birth complications such as shoulder dystocia, time of delivery is crucial. Most guidelines suggest that pregnant women whose GDM is diet-controlled should be delivered at 39-40 weeks of gestation while those with poor control need to be delivered at 37-38 weeks, though the timing may vary based on individual factors, including glycemic control and fetal growth [36].

Postpartum care

GDM women should have close follow-up management for the development of chronic diabetes. ADA (2023) recommends a two-hour 75 OGTT at 4-12 weeks after delivery to check whether diabetes has remitted or the mother acquires T2DM, and once-a-year screening for diabetes, as 30-50% develop into T2DM in 10 years [9,37]. In addition, breastfeeding has been found to reduce maternal T2DM risk by 32%. Postpartum care also involves follow-up in the scenario of women with pre-gestational diabetes in order to treat their diabetes and prevent its complications in the long run. Lifestyle modification education for the reduction of T2DM is also a part of postpartum care [38].

## Conclusions

The clinical guidelines on the management of diabetes play a crucial role in optimizing maternal and fetal outcomes by providing evidence-based recommendations. Early diagnosis, tight glycemic control, and follow-up during pregnancy and into the postpartum period are underlined in the guidelines. If healthcare providers adhere to such recommendations, complications of gestational and pre-existing diabetes would be considerably minimized. However, as our knowledge of diabetes in pregnancy evolves, further research will always be needed to continue to refine these guidelines. Besides, health disparities must be addressed to make sure all women, irrespective of their background or access to health care, get the best possible care. In addition, the recommendation for all insurance providers to cover continuous glucose monitoring and checkups prior to conception may ensure equity of access to preventing diabetes and best management. It thus behooves sustained efforts to improve the response to diabetes management and support pregnant women with diabetes as one way of ensuring better maternal and child health in developing countries.
